# Dynamics of a deep-water seagrass population on the Great Barrier Reef: annual occurrence and response to a major dredging program

**DOI:** 10.1038/srep13167

**Published:** 2015-08-17

**Authors:** Paul H. York, Alex B. Carter, Kathryn Chartrand, Tonia Sankey, Linda Wells, Michael A. Rasheed

**Affiliations:** 1 Centre for Tropical Water & Aquatic Ecosystem Research (TropWATER), James Cook University, Cairns Qld, Australia; 2 Jacobs Group, 32 Cordelia St, South Brisbane, Queensland 4101, Australia

## Abstract

Global seagrass research efforts have focused on shallow coastal and estuarine seagrass populations where alarming declines have been recorded. Comparatively little is known about the dynamics of deep-water seagrasses despite evidence that they form extensive meadows in some parts of the world. Deep-water seagrasses are subject to similar anthropogenic threats as shallow meadows, particularly along the Great Barrier Reef lagoon where they occur close to major population centres. We examine the dynamics of a deep-water seagrass population in the GBR over an 8 year period during which time a major capital dredging project occurred. Seasonal and inter-annual changes in seagrasses were assessed as well as the impact of dredging. The seagrass population was found to occur annually, generally present between July and December each year. Extensive and persistent turbid plumes from a large dredging program over an 8 month period resulted in a failure of the seagrasses to establish in 2006, however recruitment occurred the following year and the regular annual cycle was re-established. Results show that despite considerable inter annual variability, deep-water seagrasses had a regular annual pattern of occurrence, low resistance to reduced water quality but a capacity for rapid recolonisation on the cessation of impacts.

Seagrasses are highly productive, meadow-forming marine angiosperms that provide ecosystem services (e.g. nutrient cycling, sediment stabilisation, carbon sequestration and enhancement of fishery production) of great economic value^1^,^2^. Mapping and monitoring of seagrass systems over decadal time-scales has revealed alarming declines in global coverage, with habitat loss estimated at 110 km^2^ year^−1^ and accelerating^3^. The majority of the global information on seagrass change has been focused on shallow coastal populations with comparatively little known about the dynamics of deeper offshore seagrasses (>15 m depth), except for the Mediterranean *Posidonia oceanica*^4^. This is despite recent information showing that deep-water seagrass meadows are extensive^5^ and highly productive^6^.

For seagrasses much of the loss has been attributed to the development of coastal watersheds that result in increased nutrients and declining water quality^7^,^8^. Seagrasses are extremely vulnerable to these changes because they are highly light dependent, requiring levels of irradiance one to two orders of magnitude higher than most marine macro-algae^9^. Global assessments of the threats to seagrasses have also identified urban and port infrastructure development and dredging as key threats^10^,^11^.

The tropical Indo-Pacific bioregion contains some of the largest and most diverse seagrass meadows on earth^10^,^12^. Located central to this bioregion, the Great Barrier Reef (GBR) protects large areas of the east coast of Queensland, Australia from wave exposure, providing habitat for seagrass in the lagoon between the reef and the mainland^13^. Seagrasses in this region occur in estuarine, coastal, reef and deep-water habitats^14^. Estuarine and coastal habitats situated in shallow water nearest to population centres have been studied relatively thoroughly compared to deep-water habitats where large knowledge gaps persist^5^,^13^,^14^.

Recent estimates of deep-water seagrass coverage in the GBR place them among the world’s most extensive, covering approximately 32,000 km^2^ and accounting for over 90% of total seagrass cover in the region^5^,^12^. Like their coastal and estuarine equivalents these meadows also occur in proximity to major population centres and areas where threats from coastal and port and shipping developments are common^13^.

Deep-water seagrass (>15 m depth) in the GBR consists of five species from the *Halophila* genus (*H. ovalis*, *H. decipiens*, *H. spinulosa*, *H. tricostata* and *H. capricorni*)^5^. These *Halophila* assemblages also grow at shallower depths when the optical properties of the water (e.g. low light levels in turbid environments) align with those typically found in clearer waters at greater depths^15^. *Halophila* species are structurally small and have low biomass relative to other seagrass species, which means they have lower respiratory demands on photosynthetic carbon and therefore lower light requirements for growth and survival^16^,^17^,^18^,^19^. This allows *Halophila* to survive at greater depths than other species. The ecological trade-off for this low biomass is that they have limited carbohydrate stores to support them once light levels drop below their requirements^20^. *Halophila* species have been shown to have much lower resistance to light deprivation than other seagrass species with mortality occurring in days to weeks rather than months^20^,^21^. To compensate for low resistance to light stress and seasonal variability in water quality, *Halophila* species rely on sexual reproduction and the dispersal of seeds to recover quickly once a disturbance or stress has abated^22^,^23^,^24^. This ability to recolonise quickly after disturbance has resulted in *Halophila* being referred to as ‘ruderal, or colonising’ species^22^,^25^,^26^.

The dynamic nature of deep-water seagrass populations and their intimate coupling to environmental conditions creates problems for monitoring and mapping these meadows because the abundance and distribution of seagrass may vary substantially both spatially and temporally. Consequently long term studies of their dynamics are rare and predominately confined to offshore waters in the Caribbean and the east coast of the United States where annual or highly seasonal life cycles have been reported^22^,^26^,^27^.

In the GBR region there has been considerable recent focus on the potential impacts of ports and shipping developments on the values of the Great Barrier Reef Marine Park (GBRMP) including seagrasses^13^,^28^. There is strong evidence from around the world that dredging can have impacts on seagrasses through direct removal of plant material, reduced light availability due to sediment plumes and burial of plants by sediment from the deposition of dredge material^29^. These studies have been exclusively from shallow seagrass populations with no previous published studies on the impacts of dredging activities on deep-water seagrasses. Dredging and shipping activity adjacent to seagrass areas does not always necessarily result in significant impact^29^,^30^,^31^ with the result depending on the nature of the dredging including intensity and duration as well as the varying resistance of different seagrass populations to disturbance. Literature suggests that deep-water *Halophila* are likely to decline rapidly in response to dredging related pressures and a reduction in light availability^20^,^21^ and recover quickly from such impacts in around two years^15^,^32^. Similarly they are likely to have a low initial survival from sediment burial beyond 4 −5 cm^25^,^29^ but observations of *Halophila* recolonising spoil grounds in the tropics are relatively common^33^.

The expansion of a port facility at Hay Point in the central Queensland section of the GBR with associated large scale dredging program in 2006 provided a unique opportunity to examine some of these questions directly. We were able to monitor the abundance and distribution of these deep-water seagrass habitats seasonally over several years before, during, and after a major dredging impact event. The purpose of the monitoring was to study: (i) the seasonal and interannual dynamics of deep-water seagrass distribution and biomass; (ii) their resistance to environmental stress caused by the effects of dredging, particularly the impacts of spoil disposal and turbid dredge plumes; and (iii) their capacity for recovery from disturbance such as dredging and storms.

## Results

**Figure 1:**
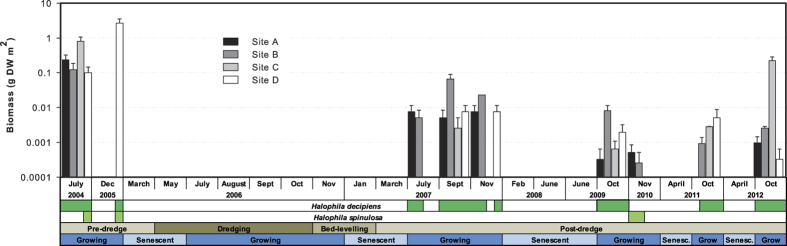
Seagrass biomass (mean ± 1 S.E.) across four sites at Hay Point from July 2004 to October 2012 in months when seagrass sampling occurred. Horizontal bars below the x-axis indicate the presence of *Halophila decipiens* (dark green) and *Halophila spinulosa* (light green). Dark and medium brown horizontal bars below the x-axis denote the period of dredging and bed-levelling respectively while light brown bars indicate the pre- and post-dredging period. Dark blue horizontal bars below the x-axis represent the growing season for seagrasses in the region while pale blue bars represent the seagrass senescent season. Note log-scale on y-axis.

Seagrass presence was highly seasonal with an annual occurrence between July and December of each year, apart from 2006 during dredging when no seagrass occurred (Fig. 1). Two species of seagrass (*H. decipiens* and *H. spinulosa*) were identified during the monitoring period (2004–2012). *Halophila decipiens* was the more common of the two species and was recorded at most sites between July and December each year, except in 2006 during the dredging program and in November 2010. *Halophila spinulosa* was present only at site D before dredging occurred and then in very low abundance at sites A and B in November 2010 (Fig. 1).

Analysis of seagrass biomass also found a significant two-way interaction between growing season and disturbance phase (PERMANOVA—*Pseudo F* = 2.691, *p* = 0.0001, Table 1). Biomass was significantly higher in the growing season before dredging occurred than during the post dredging phase where average biomass was only 1% of pre-dredge levels, with a peak of 12% of pre-dredge levels in October 2012. Both of these periods had significantly greater biomass than in the dredging period, when no seagrass was present during the growing season (pairwise comparisons—*p* < 0.01; Fig. 1). Seagrass was absent in every senescent season sampled (Fig. 1). A significant interaction occurred between site and time which was nested in both growing season and dredging phase (PERMANOVA—*Pseudo F* = 7.682, *p* = 0.0001, Table 1) however there was no significant effect of site or significant interactions with site and either growing season or dredging phase (Table 1).

**Figure 2:**
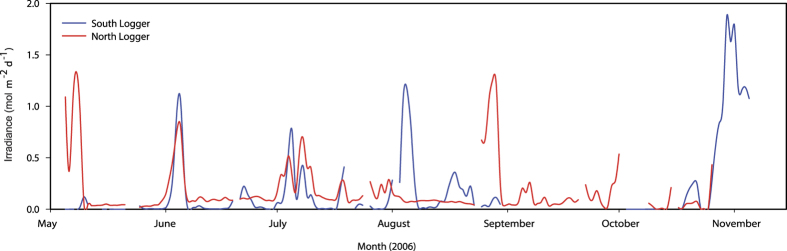
Benthic total daily irradiance (photosynthetically active radiation) during the dredging period at the north and south logger sites.

Daily irradiance at both of the light logger sites was extremely low, often < 0.5 mol m^−2^ d^−1^, for much of the dredging period, with several peaks of ~1.2 mol m^−2^ d^−1^ for several days in succession (Fig. 2).

## Discussion

Over the 8 year study period deep-water seagrass meadows had an annual cycle of occurrence being present only from July to December each year. *Halophila decipiens*, the dominant species in the study, has a global, pan-tropical distribution and studies of deep-water meadows indicate an annual habit may be common throughout the range^22^,^23^,^26^,^34^. *Halophila spinulosa* at Hay Point was a marginal species and was only detected during three of the twenty three sampling periods and at individual sites on each occasion. The dynamics of *H. spinulosa* are poorly studied, however this species is structurally more complex and has a greater biomass than other *Halophila* species and has been shown to survive longer periods of low light stress than congeneric species^35^. While our study demonstrates an annual occurrence of *Halophila* in the region, additional information on germination, growth, flowering and seed development would be required for confirmation of an annual lifecycle. It is also likely that seasonality for deep-water seagrass may vary with site or latitude as another recent study approximately 180 km to the north has found, that while deep-water *Halophila* populations were highly seasonal they maintained a continued presence throughout the year^15^.

Annual, seasonal and ephemeral life history traits in plants is thought to be a strategy for persistence in variable environments such as in desert and semi-arid terrestrial ecosystems^36^. Variation in deep-water seagrass abundance is likely to be driven by strong seasonal differences in climate including rainfall, high river flow from proximal catchments, wind patterns, and ensuing chronic turbidity impacting on light availability. In particular, peak rainfall during the wet season (December/January–April) leads to reduced water quality and irradiances and reduced wind from July to November enhances the deep-water light environment through a reduction in sediment resuspension. Both *H. decipiens* and *H. spinulosa* are considered to be physiologically suited to low light environments with a minimum light requirement of 4.4–8.8% of surface irradiance^37^. However, while low biomass is an advantage for survival in marginal light environments due to lower demands on respiratory carbon, it leaves them with lower levels of stored carbohydrates and therefore these species have low resistance to disturbance such as extreme or chronic low light stress from high turbidity events^21^. Studies have found that complete shading, such as a prolonged increase in turbidity, can stimulate changes in the architecture and growth characteristics within 9–14 days and will cause rapid decline in structurally small species like *H. decipiens* after approximately 30–40 days^21^,^38^. A study of tropical, reef-front seagrass assemblages in Malaysia (dominated by *Halophila* species including *H. decipiens* at the deeper seaward edge) concluded that the reduction of light by monsoonal winds and rainfall were a major driver of patterns of abundance^39^.

Water temperature is another variable that has the potential to affect seasonal biomass of deep-water seagrasses at Hay Point given the relatively large annual range (19–30 °C). It is unlikely however, that temperature alone could explain the absence of seagrass in the senescent season given the presence of *H. decipiens* in warmer thermal environments in lower latitudes^5^ and also in cooler temperate waters^34^.

Our study also indicates that these seagrass communities are sensitive to large-scale dredging impacts. The turbidity created during the capital dredging program in 2006 led to persistent low light conditions at the site. Seagrass was present during the growing season every year of the study except during the dredging period in 2006. This absence cannot be explained by any abnormal local or regional climate events. Seagrass recovered quickly after the disturbance had abated with the regular seasonal recruitment re-established in the year following dredging. However despite a return of seagrass, post dredging biomass failed to reach pre-dredge levels in the six years since completion of the capital dredging. Surveys across the entire GBR in the late 1990’s indicate that deep-water seagrasses in the region that incorporates the study area were generally less abundant than our pre-dredge level^5^. This may be a factor of long-term climate cycles and the switch from low to average or above-average rainfall patterns in 2007. This indicates a baseline of two years prior to disturbance for highly variable species such as *Halophila* is unlikely to be adequate and may have caught the seagrass at a peak of its long term (decadal) distribution^40^.

The lack of seagrass during the growing season in 2006 while dredging was being conducted is likely to be due to the extremely low light levels throughout this period from the turbidity plume. Unfortunately, as the footprint of the dredge plume was larger than original models predicted and extended beyond the anticipated control sites it was not possible to definitively demonstrate this impact. However, daily dose measured from the two light loggers at the site were extremely low for much of the six-month dredging period with only short peaks of around 1.3 mol m^−2^ day^−1^ often approaching zero for periods of greater than a week in duration. These values are likely to be well below the light required to sustain these species^35^.

Our study examined the impacts of a very large capital dredging campaign that extended for more than 8 months with up to 9 million m^3^ of material dredged and disposed at the spoil ground. It is worth noting that several other much smaller (in volume or duration) maintenance and capital dredging programs have been conducted in the port and these did not result in extensive or persistent plumes interacting with seagrass meadows (150,000 m^3^ maintenance in 2008; 59,000 m^3^ small capital works in 2008; 275,000 m^3^ dredging for additional loading berths in 2010/11). This illustrates the variety in impact outcomes from different dredging programs. It is not just intensity and duration that can lead to these differences; there is an enormous range of dredging techniques and equipment that strongly influence the amount of turbidity generated from dredging^29^,^41^. The smaller capital programs in Hay Point used a variety of dredging techniques including backhoe dredging that produces only very localised and small scale plumes as opposed to the large trailer suction hopper dredge used as part of the capital program in 2006^41^. These differences in dredging are not confined to our study site but commonly occur throughout the world where coastal dredging occurs, and will be a key consideration in determining impact to seagrass.

The rate of recovery following a large disturbance event depends on factors such as the magnitude of the disturbance, the species of seagrass affected, the physical and environmental conditions of the affected area and the existence of seed banks that may aid recovery^14^. The disturbance created by the 2006 capital dredging program at Hay Point was of high magnitude with greatly reduced light levels, and prolonged chronic exposure (6 months of dredging followed by 3 months of bed-levelling). Initial seagrass recovery however, was quite fast with seagrass returning in the following growing season, firstly only 6 months after completion of the works at two sites and within 8 months at all sites, including where spoil material was deposited. *Halophila decipiens* relies predominately on sexual reproduction and is known to flower prolifically throughout the year^23^ and have a large and persistent seed bank at some locations^22^,^42^. Seeds of *H. decipiens* can remain dormant for at least two years in low light conditions and germinate rapidly when suitable light conditions return^16^,^43^,^44^. The high fecundity and rapid rate of rhizome growth makes this species well-suited to high-disturbance environments^45^. Deep-water seagrass beds 200 km to the north showed much greater capacity to recover from loss due to a series of heavy rainfall events in the La Niña of 2010/11 and Tropical Cyclone Yasi in 2011 compared to larger coastal species^15^. *Halophila* species also have the potential to reproduce and disperse asexually through vegetative fragmentation with dispersal possible over short distances^46^. The seed bank and sexual reproduction were not assessed at Hay Point as part of our study but we recommend that future programs incorporate such measures given the likely high reliance on reproductive success for long term maintenance of annual populations of deep-water seagrass.

Deep-water seagrasses in the GBR lagoon near Hay Point appear to have an annual lifecycle with a growing season from July to December. This pattern of growth may be regional, and in fact a study to the north has documented populations that are continual throughout the year although still highly seasonal in their abundance^15^. To determine the seasonal dynamics across the broader GBR deep-water seagrass populations, regular surveys across a latitudinal gradient would be required to investigate whether the same seasonal patterns of abundance occur in more northerly and less variable climates, and also along a gradient from inshore to offshore to investigate the diminishing influence of run-off and river discharge.

The ephemeral and seasonal nature of the deep-water seagrasses off the coast of Queensland has implications for management and impact assessment of dredging and development operations. In line with current industry best practice, site-specific evaluations of deep-water benthic habitats such as seagrasses within the GBR and associated environmental drivers such as light and turbidity should be conducted for as long as possible before the anticipated activities to establish appropriate impact thresholds and take into account the natural variability of local environmental conditions^29^,^47^,^48^. This study has highlighted that the timing of this monitoring is critical to capture seasonal trends and particularly target the growing season when seagrass abundance is at its highest. Furthermore, when good long-term background data is not available, benthic habitat should be assessed on their potential to support deep-water seagrass meadows by monitoring of sediments for viable seed banks and incorporating tools such as habitat suitability models^49^,^50^ into adaptive management plans. This study has also highlighted how careful selection of multiple reference sites that are isolated from the impacts is required to better detect the effects of environmental disturbance^51^. Finally, we suggest that dredge management plans should consider the senescent season of annually occurring seagrass populations as an environmental window for the timing of large capital dredging events to reduce their impact^52^.

## Methods

### Site

**Figure 3:**
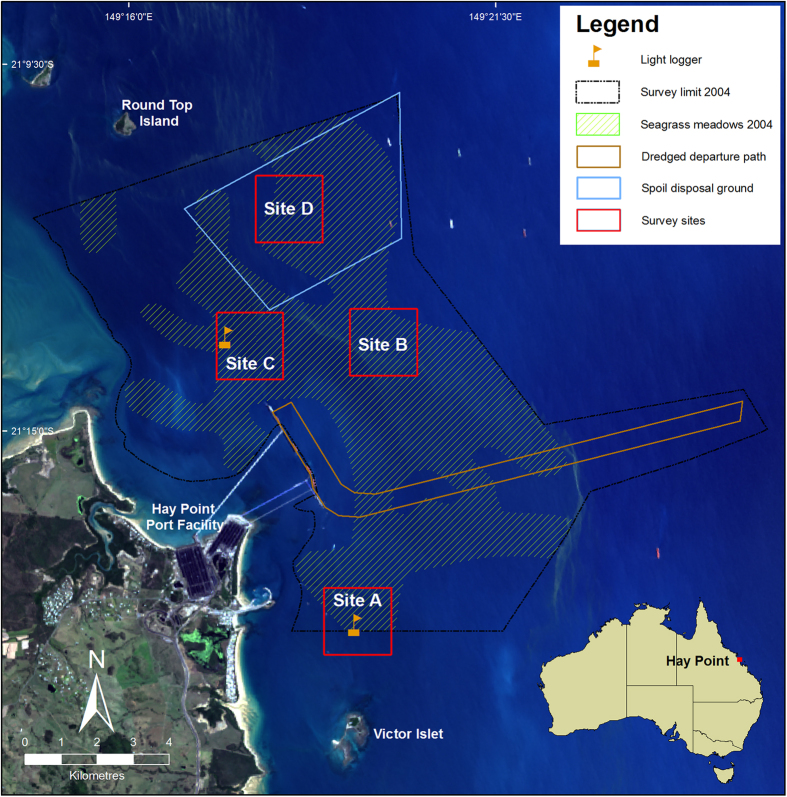
Map of the survey area showing seagrass distribution in the initial area of survey, the dredging area for the departure path, spoil ground and the seagrass monitoring sites. Map constructed in ESRI ArcMAP 10.1. Base image used is Landsat imagery courtesy of NASA Goddard Space Flight Center and U.S. Geological Survey.

Hay Point is located on the coast of central Queensland, Australia (Fig. 3), approximately 40 km south of Mackay (21°15′ S, 149°19′ E). The area is home to a commercial port that is one of the largest coal export facilities in the world with a total throughput of 108.3 million tonnes of coal in 2013/14 (http://www.nqbp.com.au/hay-point/). The last decade has seen significant development of the port and port facilities, with plans to continue expansion to accommodate the growing industrial base and increasing numbers of bulk carriers visiting the port. To accomplish this, the port authority conducted capital dredging for a new departure path and apron area at the port and established a new sea disposal site for the resulting dredged material. Dredging commenced on the 9th of May 2006 and ceased on the 17th of October 2006, with further bed levelling conducted through to the 13th of February 2007. Total volume of material removed and placed at the disposal site was approximately 9 million m^3^.

**Figure 4:**
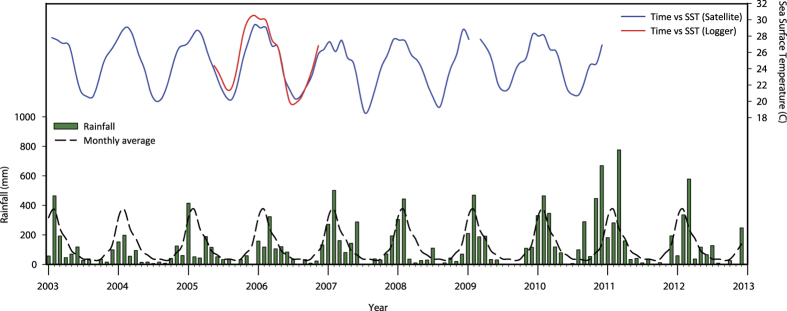
Rainfall and sea surface temperature beginning one year prior to the commencement of dredging until the end of 2012. Sea surface temperature was only available through POET until December 2010 and from loggers deployed by the Australian Institute of Marine Science from May 2005 to November 2006.

Climate in the study region consists of a distinct wet season (December–May) with large rainfall events occurring predominately from December to March followed by a dry season (June–November). Rainfall records from 2003–2013 demonstrate dry to average conditions in the period leading up to the commencement of the monitoring through to 2010, followed by a year of above average rainfall including storm and flood events from late 2010 through to early 2012 (Fig. 4). Sea surface temperature varied seasonally, generally peaking around 28–29 °C in the austral summer (December–February) and reaching average minimum temperatures of 19–21 °C in winter (June–August) (Fig. 4).

### Sampling design

Deep-water seagrasses were sampled on 23 occasions between 2004 and 2012 in the Hay Point area (Fig. 3). A baseline seagrass survey was conducted in June 2004 that resulted in the mapping of seagrass distribution, species composition and biomass. From this survey, sampling sites were established in December 2005 to monitor the effects of the capital dredging program in 2006 and to investigate seasonal and inter-annual dynamics in seagrass. Selection of sites was also influenced by the initial pre-dredge hydrodynamic modelling of the predicted dredge plume with a design intended to incorporate an inshore reference site (Site A; ~10 m depth below mean low water level [MLW]), an offshore reference site (Site B; ~15 m MLW), an inshore site impacted by the dredge plume (Site C; ~16 m MLW), and an offshore site impacted by the dredge spoil (Site D; ~18 m MLW) (Fig. 3). Seagrass assemblages at all sites consisted entirely of *Halophila* species that characterise habitats in low light environments and dominate the area affected by the dredging (over 90% of the total seagrass cover)^53^. The actual impacts, however, were much wider than anticipated by initial modelling and encompassed all of the seagrass monitoring sites^54^ and therefore sites were treated as randomly occurring within the dredge plume. Sampling occurred at three times prior to commencement of dredging and then at approximately monthly intervals during the dredging period. After dredging finished sampling was continued haphazardly with the objective of at least twice yearly surveys during the growing season for seagrasses in the area (July to December) and another in the senescent period (January to June). This pattern was not achieved in 2008 or 2010.

### Data collection—seagrass

Seagrass was sampled along nine randomly-placed 100 m transects within each site at each sampling time. The start and finish of each transect was recorded using a Global Positioning System (GPS) accurate to ±5.0 m. In order to reliably assess seagrass characteristics, an underwater CCTV camera was towed along the transect behind a vessel at drift speed (less than one knot) to recorded images of the seabed^5^. Footage was observed on a TV monitor and recorded for later processing. The camera was mounted on a sled that incorporated a net 600 mm width and 250 mm deep with a 10 mm-mesh aperture. Surface benthos including seagrass was captured in the net and used to confirm taxonomy of seagrass observed on the monitor. This technique ensured a large area of seafloor (~60 m^2^ per transect) was sampled at each site so that patchily distributed marine plant habitats were effectively measured.

Seagrass species composition and above-ground biomass were determined from ten randomly-selected frames from the video footage of each transect using a modified “visual estimates of biomass” technique^55^. The video was paused at each of the ten random time frames then advanced to the nearest point on the tape where the bottom was visible and the sled was stable on the bottom. From this frame, an observer recorded an estimated rank of seagrass biomass and species composition. To standardise biomass estimates, a 0.25 m^2^ quadrat scaled to the video camera lens was superimposed on the screen. Where seagrass was present in the sled net but not visible in transect video footage, the lowest biomass rank (0.05) was assigned to one of the 10 ranks for the transect. On completion of the video analysis, the video observer ranked five additional quadrats that had been previously filmed for calibration. These calibration quadrats were captured by a stationary camera, and then harvested, dried and weighed. A linear regression (R^2^ = 0.98, Fig. S1) was calculated for the relationship between the observer ranks and the actual harvested value. This regression was used to calculate above-ground biomass for all estimated ranks made from the survey sites. Biomass ranks were then converted into above-ground biomass estimates in grams dry weight per square metre (g DW m^−2^). Seagrass species composition was determined from sled samples and video footage according to Kuo and McComb^56^.

### Data collection—environmental

Rainfall data was collected from the nearest weather station at Mackay Aero (Station no. 33045) and sourced from publically available data (Australian Bureau of Meteorology— www.bom.gov.au). Sea surface temperature (SST) for the sampling area were estimated from moderate-resolution imaging spectroradiometer (MODIS) data collected by NASA terra-satellites and sourced through the online Jet Propulsion Laboratory Physical Oceanography DAAC Ocean ESIP Tool (POET— http://poet.jpl.nasa.gov/). Monthly averages were calculated from 2003–2010 using night-time images and a 4 km spatial resolution. To corroborate SST from satellite imagery, results were compared to publically available measurements from a water temperature logger that was stationed on the reef slope at Hay Point at a depth of 5 m between May 2005 and November 2006 (Australian Institute of Marine Sciences 2013— http://data.aims.gov.au/aimsrtds/datatool.xhtml).

Total daily irradiance (photosynthetically available radiation, PAR, measured in mol m^−2^ d^−1^) was measured using continuous PAR loggers from the commencement of dredging until a month after the dredging finished at two sites (North and South) affected by the dredge plume in 2006 (Fig. 2).

### Statistical analysis

Differences in above-ground seagrass biomass among sites (random—four levels—sites A, B, C and D, n = 9), season (fixed—two levels—growing and senescent), with respect to dredging operations (fixed—three levels—pre, during and post dredging) and time (random—nested within season and dredging stage) were tested using a four-way design of permutational multivariate analysis of variance (PERMANOVA) using the PRIMER-v6 statistical software package with the PERMANOVA+ add on^57^. This approach was chosen as the data contained many zero values and even when transformed could not fit the assumptions of normality and homogenous variance required for traditional parametric analysis of variance. Analysis was run using untransformed data using univariate PERMANOVA tests analysed from Euclidean distance matrices with 4999 unrestricted permutations of raw data^58^. When significant differences were found among treatments, pairwise comparisons were also conducted to investigate significant differences between means within groups.

## Additional Information

**How to cite this article**: York, P. H. *et al.* Dynamics of a deep-water seagrass population on the Great Barrier Reef: annual occurrence and response to a major dredging program. *Sci. Rep.* **5**, 13167; doi: 10.1038/srep13167 (2015).

## Supplementary Material

Supplementary Information

## Figures and Tables

**Table 1:** PERMANOVA results for four-way analysis of seagrass biomass across sites, through disturbance phases (DP), between seasons and among times nested in both disturbance phase and seasons.

SOURCE	df	MS	Pseudo-F	P(perm)
**Site**	3	1.338	2.193	0.2149
**Season**	1	3.674	2.907	**0.0001**
**Disturbance phase (DP)**	2	2.453	2.691	**0.0002**
**Site × Season**	3	1.338	2.193	0.2055
**Site × DP**	6	1.003	1.643	0.2084
**Season × DP**	2	2.453	2.691	**0.0001**
**Time [Season × DP]**	17	2.308	0.136	1.0000
**Site × Season × DP**	6	1.003	1.643	0.2134
**Site × Time [Season × DP]**	51	0.610	7.682	**0.0001**
**Residual**	736	0.0794		
**Total**	827	128.02		
