# Supplementary material for: Dynamics of a deep-water seagrass population on the Great Barrier Reef: annual occurrence and response to a major dredging program
Source: Sci Rep. 2015 Aug 17;5:13167. doi: 10.1038/srep13167 (PMC4538371; doi:10.1038/srep13167)

Dynamics of a deep-water seagrass population on the Great Barrier Reef: annual occurrence and response to a major dredging program

Paul H.York[1], Alex B. Carter[1], Katie Chartrand[1], Tonia Sankey[1], Linda Wells[2], Michael A. Rasheed[1]*

[1]Centre for Tropical Water & Aquatic Ecosystem Research (TropWATER), James Cook University, Cairns Qld, Australia

[2]Jacobs Group (Australia), South Brisbane, Qld, Australia

*Corresponding author: Dr Michael Rasheed

P.O Box 6811, Cairns QLD 4870 Australia

Tel: + 61 7 4232 2010

E-mail: michael.rasheed@jcu.edu.au

Supplementary Figure 1: Linear regression used to convert observer visual rank from CCTV footage into biomass estimates.

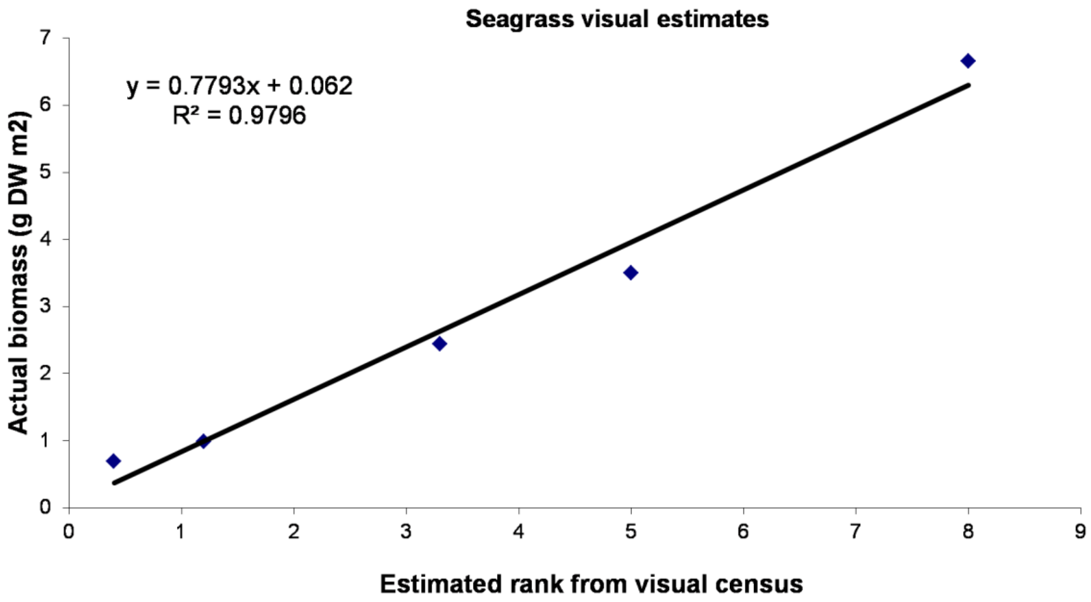

Supplement: Supplementary Information [file srep13167-s1.pdf]
